# Construction of Learning Pathways and Learning Progressions for High School English Reading Comprehension Based on Cognitive Diagnostic Assessment

**DOI:** 10.3390/jintelligence13110140

**Published:** 2025-11-04

**Authors:** Fei Wang, Zhaosheng Luo, Ying Miao, Shuting Zhou, Lang Zheng

**Affiliations:** 1 School of Psychology, Jiangxi Normal University, Nanchang 330022, China; sofi@jxnu.edu.cn (F.W.);; 2 School of Humanities and Media, Wenshan University, Wenshan 663099, China; 3 School of Education, Jiangsu University of Technology, Changzhou 213001, China

**Keywords:** cognitive diagnostic theory, item response theory, learning pathway, learning progression, English reading comprehension

## Abstract

To meet the growing demands for competency-based and personalized instruction in high school English reading, this study investigates a quantitative approach to modeling learning pathways and progressions. Traditional assessments often fail to capture students’ fine-grained cognitive differences and provide limited guidance for individualized teaching. Based on cognitive diagnostic theory, this study analyzes large-scale empirical data to construct a progression framework reflecting both the sequencing of cognitive skill development and the hierarchical structure of reading abilities. A Q-matrix was calibrated through expert consensus. A hybrid cognitive diagnostic model was used to infer students’ knowledge states, followed by cluster analysis and item response theory to define progression levels, which were mapped to national curriculum standards. The findings reveal that students’ mastery of cognitive attributes follows a stepwise developmental pattern, with dominant learning trajectories. The constructed learning progression aligns well with curriculum-based academic quality levels, while uncovering potential misalignments in the positioning of some skill levels. Students with identical scores also showed significant variation in cognitive structures. The proposed model provides a data-informed foundation for adaptive instruction and offers new tools for personalized learning in English reading comprehension.

## 1. Introduction

Amid the global transformation of educational systems, the prevailing philosophy of instruction is undergoing a fundamental shift from traditional knowledge transmission to competency-based education. Central to this transformation is a growing emphasis on student-centered, personalized, and precision teaching approaches. This paradigm aligns closely with UNESCO’s global agenda for educational equity and quality (UNESCO 2015), which advocates a learner-focused approach that respects individual differences and fosters holistic and personalized student development.

In line with this global trend, the Ministry of Education of China has issued the General Senior High School English Curriculum Standards (Ministry of Education of the People’s Republic of China 2020), which emphasize the development of students’ cognitive abilities in acquiring, processing, analyzing, and applying information through English. English reading comprehension, as a critical indicator of students’ overall English proficiency, is identified as a central component. The Curriculum further underscores the need to establish a common foundation for English learning in high schools, address individual learning needs, and promote task-based language learning, thereby calling for a more refined understanding and guidance of students’ learning processes.

However, traditional educational assessment frameworks, such as Classical Test Theory (CTT) and Item Response Theory (IRT), focus primarily on evaluating students’ overall proficiency, typically yielding a single score to stratify learners. While such approaches may serve broad screening purposes, they often lack diagnostic granularity. Consequently, teaching interventions based on these results tend to lack specificity, making it difficult to realize the personalized and differentiated instruction envisioned by the new curriculum standards.

In this context, Cognitive Diagnostic Theory (CDT) has emerged as a new generation of psychometric model designed to address the limitations of traditional assessments. Rooted in cognitive psychology and educational measurement, CDT offers a fine-grained analysis of students’ mastery over specific cognitive attributes. For instance, Templin and Henson (2010) elaborated on how CDT operationalizes a Q-matrix to map items onto latent attributes, enabling detailed diagnostic inferences about students’ cognitive strengths and weaknesses. By transforming abstract test scores into actionable profiles of attribute mastery, CDT supports targeted remediation and individualized instruction. Moreover, it provides an empirical foundation for constructing learning pathways and learning progressions, thereby offering theoretical support for the design of personalized learning and adaptive learning systems (Leighton and Gierl 2007a).

The following research questions guide this study:

1. What are the core cognitive attributes underlying high school English reading comprehension, and how can they be stably measured using cognitive diagnostic modeling?
2. What is the dominant learning pathway for the acquisition of these cognitive attributes among high school students as revealed by examination data?
3. How can students’ knowledge states be stratified into a hierarchical learning progression that aligns with national curriculum standards using clustering and IRT techniques?

## 2. Literature Review

The application of cognitive diagnostic models in language assessment has gradually expanded, yielding significant outcomes. In the domain of English reading comprehension testing, numerous studies have employed cognitive diagnostic models for diagnostic purposes. For example, Kasai (1997) applied the Rule Space Model to the TOEFL reading section, identifying sixteen cognitive attributes. Jang (2009) utilized the Fusion Model to investigate the validity of the LanguEdge reading comprehension test, successfully diagnosing students’ reading skill mastery. Lee and Sawaki (2009) applied the Fusion Model, the Generalized Diagnostic Model, and the Latent Class Model to English as a Second Language listening and reading assessments. Wang and Gierl (2011) employed the Attribute Hierarchy Method to analyze cognitive skills in the Scholastic Assessment Test critical reading section. Ravand (2016) applied the Generalized Deterministic Inputs, Noisy “And” Gate model to the Iranian National University Entrance Examination reading comprehension test, validating five cognitive attributes and their hierarchical relationships, confirming the model’s suitability for language ability estimation. Kim (2015) used the Fusion Model in an adult second-language placement test in a United States university, defining ten reading cognitive attributes and generating diagnostic feedback. Cai (2010) integrated foreign research and China’s General Senior High School English Curriculum Standards (Ministry of Education of the People’s Republic of China 2020) to identify eight cognitive attributes in Chinese college entrance examination reading tasks, providing diagnostic feedback at individual and group levels. Du and Ma (2018) established eight core reading attributes via tree regression analysis. Zhao et al. (2025) applied the Generalized Deterministic Inputs, Noisy “And” Gate model to diagnose the reading abilities of nearly fifty thousand college entrance exam English test takers, identifying seven reading attributes. Luo (2019) introduced cognitive diagnostic theory to university English placement testing based on the College English Placement Test at Hunan University, constructing a reading ability cognitive model and conducting dual-level diagnostic analyses for non-English majors. Collectively, these studies reveal that examinees with identical total or reading scores often differ significantly in attribute mastery, underscoring the limitations of traditional assessments and highlighting the unique value of cognitive diagnosis, while laying a foundation for deeper applications.

Despite these advances, challenges remain. Current cognitive diagnostic model research primarily focuses on diagnosing cognitive attributes, providing profiles of learners’ strengths and weaknesses but lacking in further applied exploration. Meanwhile, the construction of learning pathways and learning progressions—core frameworks for personalized instruction—lacks objective quantitative methods and large-scale empirical data support. Within cognitive diagnosis, a learning pathway is regarded as an ordered sequence of cognitive development reflecting inclusion relations among knowledge states, resonating with Vygotsky’s Zone of Proximal Development theory, which emphasizes progressive learning based on existing abilities (Vygotsky 1978). The National Research Council (NRC 2007) defines learning progressions as empirically grounded descriptions of increasingly sophisticated ways of thinking about a domain that students can exhibit over extended periods of learning, reflecting a gradual development from novice to expert understanding. Subsequent studies conceptualize them as evidence-based, testable hypotheses about learning pathways (Corcoran et al. 2009).

However, existing studies on constructing learning paths tend to overly rely on expert judgment or qualitative methods such as observations and interviews (e.g., using think-aloud protocols to investigate test-takers’ implicit cognitive processes and strategies for each item). These approaches are inherently subjective to some extent, and the resulting models often lack stability and generalizability. Similarly, in constructing hierarchical learning progressions, researchers mainly rely on limited longitudinal tracking data or hypothetical academic standards, without objective quantification methods or support from large-scale empirical data (Xin et al. 2015). These methodological limitations lead to significant shortcomings in the scientific rigor, universality, and practical applicability of the resulting learning paths and progressions in real large-scale educational settings. Consequently, they have not fully leveraged the powerful potential of cognitive diagnosis in precision education. Therefore, how to utilize large-scale empirical data and adopt objective and quantitative methods to systematically construct English reading learning paths and progressions with cognitive sequencing and hierarchical abilities has become a critical scientific challenge in the field of language education measurement. Addressing this issue not only enhances the accuracy of personalized instruction but also provides students with clearer and more effective learning guidance.

## 3. Methods

This study aims to systematically construct a framework for English reading learning pathways and learning progressions that incorporates both cognitive sequencing and hierarchical ability levels, based on large-scale empirical analysis guided by cognitive diagnostic theory.

### 3.1. Participants and Instrument

#### 3.1.1. Participants

The data for this study were obtained from all 361,967 candidates (excluding absentees) who registered for and took the provincial English college entrance examination in a province of China in 2020. This examination is the English subject test of China’s National College Entrance Examination (commonly known as Gaokao), a high-stakes, standardized test that is mandatory for all high school graduates wishing to attend university in China. The test is open to all students who meet the eligibility requirements set by the Ministry of Education, and participation is essentially universal among high school graduates. Therefore, the participants are representative of the entire population of final-year high school students in the province who intended to pursue higher education. However, due to the confidentiality policies of the examination authority, no demographic data (such as gender, age, or other personal characteristics) are provided, and only exam scores were available for analysis. Therefore, we cannot offer a detailed demographic profile of the participants.

#### 3.1.2. Instrument

The instrument consisted of 20 reading comprehension items from the 2020 provincial English college entrance examination. Data processing and analysis were conducted using SPSS 23.0, R 4.0.5, and the flexCDMs analysis platform (Tu 2019). Instrument reliability was evaluated through two approaches: Overall test reliability: Cronbach’s α coefficient = 0.805 (>0.7 threshold), computed under Classical Test Theory. Attribute-level classification reliability: To assess the consistency of mastery/non-mastery classifications for each attribute, we followed the simulation-based method proposed by Templin and Bradshaw (2013). This method estimates the reliability of the final attribute classification for each student, which is derived from the full cognitive diagnostic model that accounts for the influence of multiple attributes on each item. As shown in Table 1, the classification reliability for all attributes exceeded the 0.7 threshold, indicating a high degree of consistency.

A preliminary 8-attribute Q-matrix was established through independent calibration by five senior English teachers (Kendall’s W = 0.51). After validation, Attribute A8 (“Main Idea”) was removed due to measurement by only two items. This decision is consistent with the methodological approach of Cai (2010), as it violates the minimum requirement of three items per attribute for reliable diagnostic identification (insufficient for model identifiability per Ding et al. 2009; Tu 2019; Xu and Zhang 2016), resulting in a finalized 7-attribute Q-matrix.

### 3.2. Procedure and Data Analysis

The analysis was conducted in three main stages. First, we selected the most appropriate cognitive diagnostic model by comparing the fit of several common models (DINA, DINO, G-DINA, etc.) using AIC, BIC, and RMSEA criteria. A mixed-model approach using the Wald test was employed to ensure both model fit and parsimony. Second, using the selected model, we estimated each student’s knowledge state (i.e., their mastery profile across the seven attributes). These knowledge states were then used to construct the learning pathways by identifying the most frequent transitional sequences. Third, we constructed the learning progression by applying K-means clustering to the knowledge states and using the 2PL IRT model to estimate an ability value for each cluster. Finally, these ability values were mapped to the three academic quality levels defined in the national curriculum standards.

## 4. Procedure and Results

### 4.1. Construction of the Q-Matrix

Cognitive attributes play a fundamental and central role in cognitive diagnostic assessment. Essentially, cognitive diagnosis aims to identify students’ mastery of specific cognitive attributes (Wu et al. 2020). These attributes represent the necessary knowledge, strategies, skills, processes, and methods required to complete tasks, providing a detailed description of the internal psychological processes involved in problem solving (Cai 2010). As emphasized by Templin and Henson (2010), cognitive attributes serve as the bridge connecting observable behaviors and latent cognitive structures, forming the cornerstone for constructing cognitive diagnostic models. Precise definition of these attributes is critical to ensuring the validity of diagnostic results and to guiding instructional practice (Leighton and Gierl 2007b).

Building on previous research and fully considering the curriculum standards, as well as the requirements of the National Matriculation English Test, Cai (2010) specifically defined cognitive attributes for English reading comprehension in the context of the Chinese college entrance examination. This study holds significant practical value as it integrates linguistic theory with the realities of Chinese education, thereby enhancing the utility of cognitive diagnosis for high school English teaching and assessment. Eight core cognitive attributes for English reading comprehension were identified, which are summarized in Table 2.

Cognitive attributes are linked to test items through the Q-matrix, which is a two-dimensional matrix that serves as a bridge between students’ observable item responses and their unobservable cognitive states. Each row of the Q-matrix corresponds to a test item, and each column corresponds to a cognitive attribute. A value of “1” indicates that the item measures the corresponding attribute, while a “0” indicates it does not. This concept was first introduced by Tatsuoka (1983) and has since been widely used in cognitive diagnostic research. The initial Q-matrix is typically constructed based on expert judgment. Although this method is commonly adopted in practice, it may introduce subjectivity, and therefore requires rigorous validation procedures to ensure its accuracy (De La Torre 2011).

In this study, 20 reading comprehension items from the 2020 provincial English college entrance examination were analyzed. Five senior high school English teachers, each with over ten years of experience, independently calibrated the cognitive attributes assessed by each item. This “back-to-back” independent rating approach was adopted to prevent influence among experts and to enhance objectivity. Inter-rater agreement yielded a Kendall’s W of 0.51. This value exceeds the established threshold of Kappa ≥0.4 used in diagnostic studies to confirm expert consensus reliability (Shi 2017; Wang 2017), providing empirical support for the Q-matrix validity. Final attribute assignments followed majority rule (≥3/5 experts), establishing the preliminary Q-matrix (Table 3).

During optimization, Attribute A8 (“Main Idea”) was excluded due to measurement by only two items (violating the 3-item minimum for reliable diagnosis), consistent with methodological precedents (Cai 2010).

In cognitive diagnosis, it is crucial to ensure that each cognitive attribute is effectively measured and distinguishable. Studies have shown that when a cognitive attribute is measured by too few items, it becomes difficult to accurately diagnose students’ mastery of that attribute. For instance, findings from Ding et al. (2009), Tu (2019) and Xu and Zhang (2016) indicate that when an attribute is measured by fewer than three items, the test lacks sufficient discriminative power for that attribute. This means that even if there are genuine differences in students’ abilities regarding the attribute, the test may fail to capture them accurately, thereby compromising the validity of the diagnostic results. These findings provide important empirical support for the construction and optimization of the Q-matrix, highlighting the necessity of ensuring sufficient measurement of each attribute in test design.

Based on this principle, the initial matrix was optimized. Attribute A8 (“Main Idea”) was measured only twice in the preliminary matrix, falling short of the recommended minimum of three items. To ensure diagnostic accuracy and reliability, this attribute was removed. Consequently, only the first seven attributes were retained. The finalized Q-matrix with seven attributes will be used for subsequent cognitive diagnostic analysis. This optimization enhanced the precision of diagnostic outcomes and ensured the validity of cognitive state descriptions, thereby better supporting personalized instruction and learning pathway construction.

### 4.2. Cognitive Diagnostic Model Selection

#### 4.2.1. Model Fit Testing

Selecting an appropriate cognitive diagnostic model is a prerequisite to accurately assessing students’ cognitive states. As highlighted by Tatsuoka (1983) and Templin and Henson (2010), cognitive diagnostic models are a class of statistical models that integrate cognitive variables to link test performance with mastery of underlying cognitive attributes. Model selection must consider theoretical assumptions and algorithmic characteristics to determine suitability for specific research contexts.

However, cognitive diagnostic models are numerous and relatively complex, making it difficult to thoroughly analyze the principles of each model and manually select the optimal one. Therefore, in practice, researchers often adopt a data-driven approach. This method analyzes and compares actual data to select the model from existing ones that provides the best fit and most accurately reflects the characteristics of the data. To obtain a model with better fit, this study utilizes the CDM and GDINA R packages to evaluate the parameters of several commonly used cognitive diagnostic models. These models include DINA (Deterministic Input, Noisy “And” Gate), DINO (Deterministic Input, Noisy “Or” Gate), RRUM (Reduced Reparameterized Unified Model), ACDM (Additive Cognitive Diagnostic Model), LCDM (Log-linear Cognitive Diagnostic Model), LLM (Linear Logistic Model), G-DINA (Generalized DINA), and Mixed Model. By comparing the parameters of these models (as shown in Table 4), the goal is to identify the cognitive diagnostic model that best fits the current high school English reading comprehension test data, thereby providing the most reliable foundation for subsequent cognitive diagnosis.

In cognitive diagnostic assessments, selecting the model that best represents the data is of paramount importance. Model fit statistics are generally divided into two levels: absolute fit statistics and relative fit statistics. The former evaluates the degree to which the model aligns with the observed data, serving as a prerequisite for all subsequent analyses by ensuring that the model can adequately explain the data in an absolute sense. The latter adopts a comparative perspective to identify the optimal model among a set of candidate models.

In this study, the absolute fit was assessed using the Root Mean Square Error of Approximation (RMSEA), a widely applied fit index in structural equation modeling and cognitive diagnostic modeling. RMSEA adjusts for model complexity by incorporating degrees of freedom and reflects how well the model reproduces the population covariance structure. According to the criteria proposed by Browne and Cudeck (1992), an RMSEA value below 0.05 indicates excellent fit; values between 0.05 and 0.08 indicate acceptable fit; and values above 0.10 suggest poor fit. Based on the results presented in Table 4, it can be concluded that all evaluated cognitive models demonstrate satisfactory absolute fit, thus providing a sound basis for subsequent model comparisons.

The process of model comparison is essentially a relative fit evaluation aiming to select the best model from among those demonstrating good absolute fit. During this process, the Akaike Information Criterion (AIC) and the Bayesian Information Criterion (BIC) are primarily considered, alongside the total number of estimated parameters and the deviance statistic. The number of parameters reflects model complexity: models with more parameters are more flexible but may overfit the training data and perform poorly on new data. Therefore, under similar fit quality, models with fewer parameters are preferred, consistent with Occam’s Razor principle, which advocates for parsimony (Jefferys and Berger 1992).

In selecting cognitive diagnostic models, AIC and BIC are the primary criteria. As noted by Vrieze (2012), models with lower AIC and BIC values demonstrate better fit because these metrics simultaneously consider goodness-of-fit and penalize complexity, thereby mitigating overfitting. Results from Table 4 clearly show that the Mixed Model yields the smallest AIC, BIC, and deviance values among the candidate models (highlighted in red). This strongly supports the superior fit of the Mixed Model relative to other models. Consequently, the Mixed Model was adopted for further analyses to ensure accurate diagnostic outcomes and robust modeling.

#### 4.2.2. Model Selection for Multi-Attribute Items

In the field of cognitive diagnosis, striking a balance between model fit and parsimony is critical. To this end, De La Torre and Lee (2013) proposed a model selection method based on the Wald test, specifically designed to identify the most appropriate parsimonious model for multi-attribute items while preserving adequate model-data fit. Multi-attribute items refer to those that assess two or more cognitive attributes. Parsimonious models, characterized by fewer parameters and more intuitive interpretability, are essential for enhancing both the practicality and comprehensibility of diagnostic outcomes. This model selection approach is commonly referred to as the Mixed Model strategy.

The core idea of this approach is as follows: for items measuring only a single attribute, the saturated Generalized Deterministic Inputs, Noisy “And” gate (G-DINA) model is applied directly. In contrast, for items involving multiple attributes, a data-driven selection process is employed to determine the most suitable parsimonious model. Following these rigorous rules, each multi-attribute item was evaluated individually, resulting in the final construction of the mixed model. For single-attribute items, the G-DINA model was used. For the 14 multi-attribute items, the Wald test was used to select the most appropriate parsimonious model. The item-level selection outcomes are presented in Table 5. For example, for Item 11, the LLM was selected (*p* = 0.2442), while for Item 7, no parsimonious model fit as well as the saturated G-DINA model, so the G-DINA model was retained. This hybrid model, combining the strengths of both saturated and parsimonious models, was validated using the Wald test to ensure that the selected parsimonious models did not significantly worsen the model fit (*p* > 0.05). This approach balances model fit and interpretability, providing a robust foundation for subsequent diagnostic estimations.

In this study, the GDINA R package was used to construct the mixed model framework. The Wald test was then applied to perform model selection for multi-attribute items. The Wald test is a statistical hypothesis test used to determine whether a parameter significantly differs from zero, or to evaluate whether imposing constraints on a model leads to a statistically significant deterioration in fit (Agresti and Hitchcock 2005). The decision rules for model selection in the mixed framework are as follows:

**Fit Difference Test:** This is the first step. If a parsimonious model does not significantly differ from the G-DINA model in terms of fit (i.e., *p* > 0.05), the parsimonious model is considered acceptable, as it achieves a simpler structure without a meaningful loss in explanatory power.

**Competitive Selection Among Parsimonious Models:** When multiple parsimonious models pass the fit difference test, the one with the highest *p*-value is preferred. A higher *p*-value indicates that the constraints imposed by the model are most compatible with the data, thereby maximizing information retention while simplifying the model.

**Parsimony Preference:** In practical scenarios requiring more straightforward interpretability, the most parsimonious model—such as DINA or DINO—may be prioritized, even if multiple models are statistically acceptable. However, it is important to note that when three or more attributes are involved, sufficient sample size is necessary (typically *n* > 1000) to avoid inflated Type I errors. This highlights the crucial role of sample size in ensuring the reliability of statistical inference (Templin and Henson 2010).

**Fallback to Saturated Model:** If all parsimonious models are rejected by the Wald test, it indicates that none can simplify the G-DINA model without a significant loss in fit. In such cases, the saturated model is retained as the final choice to ensure that the model captures the complexity inherent in the data.

Following these rigorous rules, each multi-attribute item was evaluated individually, resulting in the final construction of the mixed model. The item-level selection outcomes are presented in Table 5. These results provide the foundation for generating precise diagnostic feedback and support the subsequent construction of learning trajectories.

#### 4.2.3. Construction of Learning Pathways

The core objective of cognitive diagnostic assessment is to provide a fine-grained evaluation of each student’s mastery across various cognitive attributes based on their response data. Specifically, each attribute is assessed as either mastered (1) or not mastered (0), thereby shifting diagnostic emphasis from a single aggregate score to a multidimensional cognitive profile. Each student’s mastery status across attributes can thus be represented as a binary vector, typically denoted as $K_{i}=(\alpha_{1},\alpha_{2},\ldots ,\alpha_{m})$, where m is the number of cognitive attributes and $\alpha_{j}\in \left\{0,1\right\}$ indicates the mastery of attribute j. As Mislevy (1994) noted, this knowledge state vector is a key conceptual tool in cognitive diagnosis theory, enabling precise identification of individual learning needs and supporting the implementation of personalized instruction.

A student’s knowledge state is not determined by a simple count of correct answers but is a latent classification estimated by the cognitive diagnostic model. The model takes the student’s entire vector of item responses (the pattern of correct/incorrect answers across all 20 items) as input. Using a statistical estimation method (in this study, Maximum A Posteriori estimation), the model calculates the probability of the student belonging to each of the 2^7^ = 128 possible knowledge states. The student is then classified into the single knowledge state that has the highest posterior probability. This probabilistic approach provides a robust classification that considers the complex interplay between the student’s unique response pattern and the cognitive attributes required by each item as defined in the Q-matrix.

The construction of learning pathways is grounded in a fundamental assumption: human cognition progresses in a sequential order from basic to advanced skills. That is, students are more likely to master foundational attributes before acquiring more complex ones. This hierarchical progression is reflected in the knowledge states, where the presence or absence of each attribute follows a developmental sequence. More specifically, the order among elements of $K_{i}$ implies a nested structure among different knowledge states. This concept is closely aligned with Piaget’s theory of cognitive development and Gagné’s hierarchy of learning, both of which emphasize the necessity of acquiring foundational knowledge before progressing to higher-order competencies (Gagné 1985; Piaget 1970).

The construction procedure includes the following steps:

**Knowledge State Stratification**: First, students’ estimated knowledge states are categorized and stratified based on the number of mastered attributes. For example, a state with no attributes mastered (e.g., 0000000) is designated as Level 0; states with one mastered attribute (e.g., 1000000, 0000010) are Level 1, and so forth, resulting in eight levels from 0 to 7. This stratification provides a clear representation of students’ cognitive development stages.

**Generation of Pathway Sequences**: Given the complexity of learning trajectories, this study focuses on first-order transitions—paths between adjacent levels that differ by the mastery of a single attribute. A path is established when one knowledge state is a subset of another. For instance, the state (0110011) is a subset of (0110111) if the latter includes all attributes of the former plus an additional one (e.g., the fifth attribute). This subset-based identification mirrors the modeling logic of attribute hierarchy models and reveals the likely order in which students acquire knowledge (Leighton and Gierl 2007b). For example, in the domain of English reading comprehension among high school students, a representative learning pathway identified in this study is:

(0000000) → (0000010) → (0100010) → (0100011) → (0110011) → (0110111) → (0111111) → (1111111)(1)

**Figure 1:**
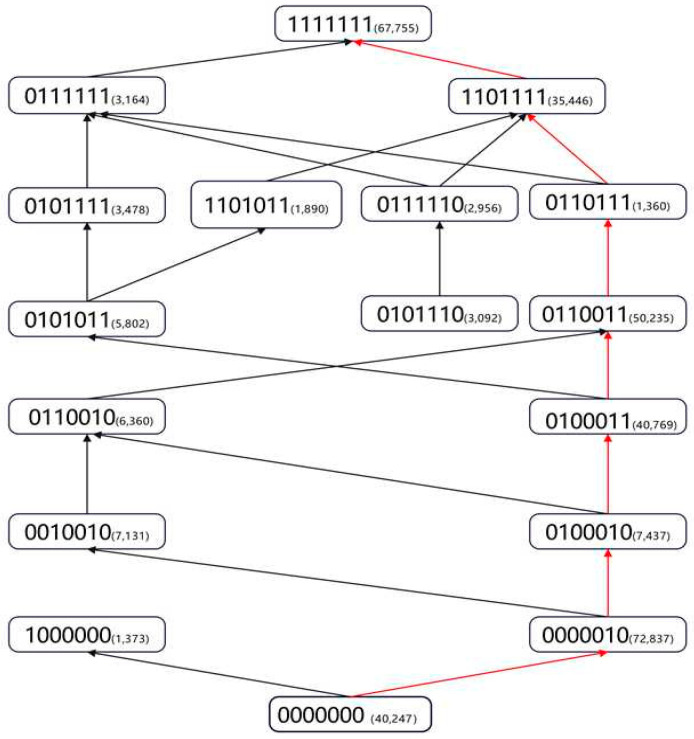
Learning Pathways in Senior High School English Reading Comprehension. Each node represents a distinct knowledge state (a binary vector of 7 attributes: A1–A7), with states stratified vertically by the total number of attributes mastered (Levels 0–7). Arrows indicate observed first-order transitions, defined as the acquisition of a single new attribute, connecting a state at one level to a state at the next higher level. It is important to note that this map represents a population-level network of probable transitions between states, not the longitudinal path of any single individual. The red path highlights the dominant (most frequent) learning trajectory.

In theory, given seven attributes, there are 2^7^ = 128 possible knowledge states. However, only 79 unique states were observed in the current dataset. To enhance analytical clarity and focus on the most prevalent developmental patterns, the subsequent pathway construction was based on the 17 most frequent knowledge states. This subset has a high degree of sample representativeness, as these 17 states collectively account for 97% (n = 351,332) of the total student population (N = 361,967). The remaining 3% of students (n = 10,635), who were distributed across 62 less frequent knowledge states, were excluded from the pathway visualization to avoid visual clutter. By linking all valid transitions between these states, a comprehensive learning pathway map for English reading comprehension was constructed. Full details of the learning pathway structure are illustrated in Figure 1. It is crucial to interpret this map as a network of possible transitions rather than a single, fixed sequence for all learners. For example, the presence of both state (0000010) (mastery of A6 only) and state (1000000) (mastery of A1 only) at Level 1 indicates that these are two distinct, alternative starting points for learners progressing from the novice state (0000000). The diagram does not imply a direct, illogical transition between these two Level 1 states where one skill is ‘lost’ and another is ‘gained.’ Rather, they represent parallel branches in the overall learning network, demonstrating that different students may begin their skill acquisition journey by mastering different foundational attributes.

**Identifying the Dominant Learning Path:** To better inform instructional practices, this study defines the most frequently observed learning path among students as the dominant learning trajectory. As illustrated by the red-marked sequence in the diagram—(0000000) → (0000010) → (0100010) → (0100011) → (0110011) → (0110111) → (0111111) → (1111111)—this dominant path is the most common trajectory found within the full sample. Specifically, a key divergence in learning occurs after the mastery of A7, where students tend to master either A3 or A4 next. Our analysis of the full dataset revealed that the path segment involving the mastery of A3 before A4, (0110011)→(0110111)→(0111111), was followed by 54,759 students. In contrast, the alternative path segment involving the mastery of A4 before A3, (0101011) → (0101111) → (1101111), was followed by 44,726 students. Because the former path is more populous, it was identified as the dominant trajectory. These findings offer clear pedagogical implications for educators and textbook developers by aligning instruction with the natural progression of students’ cognitive development.

It is important to note that the path diagram does not cover the entire student population. Several factors may explain this exclusion. First, data noise may have distorted their knowledge state estimates, potentially due to inattention, guessing, or other irregularities during the assessment. Second, in the absence of data contamination, it is possible that these students followed highly idiosyncratic learning trajectories. Since this study considers only first-order transitions—i.e., adjacent layers differing by one attribute—the framework may have overlooked more nonlinear or nonsequential learning patterns. For instance, certain students may exhibit “jumping learning,” skipping intermediate attributes and acquiring more advanced ones directly, likely due to alternative learning experiences or individual aptitude.

#### 4.2.4. Constructing the Learning Progression

While the learning path depicts the micro-level cognitive sequence based on the inclusion relations among individual knowledge states, the learning progression offers a macro-level categorization of student learning levels. By stratifying learners into hierarchical stages, the learning progression supports differentiated instruction across academic levels. To achieve this, we bridge the multidimensional, categorical output of the CDM (the knowledge states) with a unidimensional, continuous scale using Item Response Theory (IRT). It is critical to clarify the rationale for this step. The purpose of applying a unidimensional IRT model is not to argue that reading comprehension is a single latent trait, which would contradict the multidimensional premise of CDM. Rather, it is a pragmatic step to create a macro-level summary of overall proficiency. This allows us to project the complex, multi-pathway cognitive structure onto a single, interpretable scale that can be aligned with existing educational frameworks, such as the national curriculum standards, which are typically presented as a single, hierarchical progression. The construction procedure includes the following steps:

**Integration of Idiosyncratic Knowledge States**: The top 17 knowledge states covered 97% of students (*n* = 351,332). The remaining 3% (*n* = 10,635) exhibited 62 low-frequency states. To incorporate these into the progression framework, K-means clustering assigned each of these 10,635 students to existing clusters defined by the dominant 17 states. This ensured full sample inclusion while prioritizing analytical clarity over cluster validation (Chen et al. 2017; Wu et al. 2020, 2021, 2022a, 2022b). Final progression tiers derived their validity from IRT ability estimates and alignment with national curriculum standards.

**Ability Estimation**: After clustering, the study estimates students’ abilities using the two-parameter logistic (2PL) model within the Item Response Theory (IRT) framework, implemented via the mirt R package. The 2PL model, known for its balance between complexity, stability, and interpretability, provides estimates for both item difficulty and discrimination parameters, offering a precise assessment of student ability (Hambleton et al. 1991; De Ayala 2013). The average θ value for students in each knowledge state is then used to represent the ability level associated with that state.

According to Table 6, the lowest average ability score (−1.34) corresponds to the knowledge state (0000000), while the highest score (1.23) belongs to students who mastered all cognitive attributes (1111111). These estimated values provide a quantitative foundation for the stratification of the learning progression.

A noteworthy and counter-intuitive finding emerges from Table 6: the mean ability estimates (θ) for knowledge states (1000000) and (0000010) are nearly identical. This proximity is particularly striking when contrasted with the population-level mastery probabilities presented in Table 8, which identify Attribute A1 (‘Difficult Vocabulary’) as the most difficult attribute (33.3% mastery) and Attribute A6 (‘Information Matching’) as the easiest (85.8% mastery). This apparent paradox is resolved by recognizing that the θ value for a knowledge state represents the average functional ability of the students within that group, not the intrinsic difficulty of the attribute itself. The (0000010) group represents typical low-ability learners on the dominant learning path who have acquired only the most foundational skill. In contrast, the (1000000) group likely comprises a small, atypical subset of students who possess isolated vocabulary knowledge but lack the integrated comprehension strategies necessary for overall success on the assessment. The IRT model correctly assigns this atypical group a low overall ability score, as their unbalanced cognitive profile does not translate to functional reading proficiency. This finding demonstrates the model’s sensitivity in distinguishing between different types of low-performing cognitive profiles.

**Figure 2:**
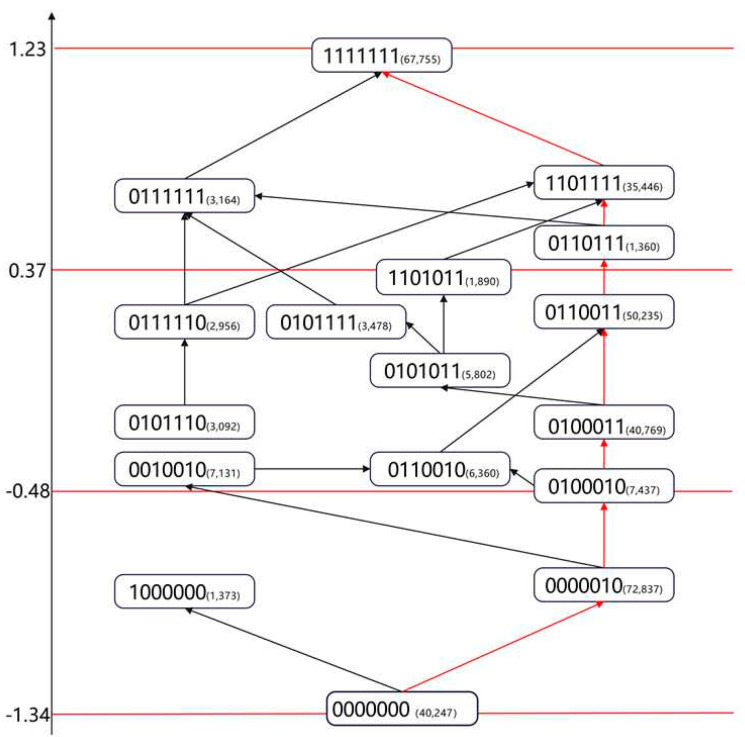
Hierarchical Levels of the Learning Progression in High School English Reading Comprehension.

**Stratification of the Learning Progression:** Academic quality standards serve as an overall description of students’ academic achievement and define the key competencies and their specific proficiency levels that students are expected to attain upon completion of different educational stages. These standards provide an authoritative reference for educational assessment. The orientation and construction of academic quality standards share strong similarities with that of learning progressions, as both aim to describe student abilities in a hierarchical manner. Therefore, the establishment of academic quality standards can draw upon the procedures used for constructing learning progressions (Xin et al. 2015). Accordingly, we aligned students’ knowledge states with the three academic quality levels defined in the national curriculum standards, classifying them into three tiers based on increasing ability values, as illustrated by the red lines in Figure 2. This approach is conceptually similar to level setting, which maps continuous ability scores onto discrete levels for better interpretability and practical application (Cizek et al. 2004). Each knowledge state was then positioned along the ability continuum according to its corresponding ability value, thereby completing the construction of the learning progression. This framework provides a clear roadmap for teachers to identify students’ current learning stages and to offer targeted instructional support accordingly.

By linking the previously identified dominant learning path—(0000000) → (0000010) → (0100010) → (0100011) → (0110011)→(0110111)→(0111111) → (1111111)—to the corresponding ability estimates, a clear trend emerges: students who master a greater number of attributes tend to exhibit progressively higher ability levels. This pattern aligns not only with established cognitive development theories but also with intuitive expectations. More importantly, this pathway represents the most common sequence of attribute acquisition among students in this study, further validating its status as the dominant learning path in the domain of English reading comprehension.

Based on the categorization of learning progression levels illustrated in the figure above, we summarized the attribute patterns within each identified level. Combined with descriptors from national curriculum standards regarding the three levels of academic achievement, we established a detailed classification of the learning progression, as shown in Table 7.

The results in Table 7 provide meaningful insights for refining the definitions of academic quality standards. For instance, Level 2 includes descriptors such as “the ability to understand conceptual vocabulary or terms using contextual clues” and “understanding the connotation and denotation of words in context.” These abilities align with attribute A1 (which involves understanding of difficult words in questions, options, or target sentences), yet our data suggests this attribute may be more difficult than previously thought, and better fits within Level 3. Similarly, Level 1 contains the descriptor “making inferences, comparisons, analyses, and generalizations based on reading or visual materials,” which was originally associated with attribute A5 (which involves drawing inferences based on textual information, context, and background knowledge; inferring word meanings; deducing author’s purpose, intention, and strategies). However, our analysis indicates this attribute is also more cognitively demanding and should be classified under Level 2.

These findings were further corroborated by students’ overall mastery probabilities for each cognitive attribute (see Table 8). The data show that students demonstrated the highest level of mastery for Attribute A6 (Information Matching), with a probability of 85.8%, followed by Attribute A7 (Complex Sentence Comprehension) at 58.7%. In contrast, students showed the lowest levels of mastery for Attribute A5 (Inference) and Attribute A1 (Difficult Vocabulary), with probabilities of 36.3% and 33.3%, respectively. These results clearly indicate that A5 and A1 are the most cognitively demanding attributes in senior high school English reading comprehension and should be classified at higher levels within the academic quality standards.

These data-driven insights provide important implications for future revisions of curriculum standards, the development of instructional materials, and the refinement of teaching strategies. They contribute to a more precise alignment between academic quality standards and students’ actual cognitive levels.

#### 4.2.5. Personalized Analysis

**Figure 3:**
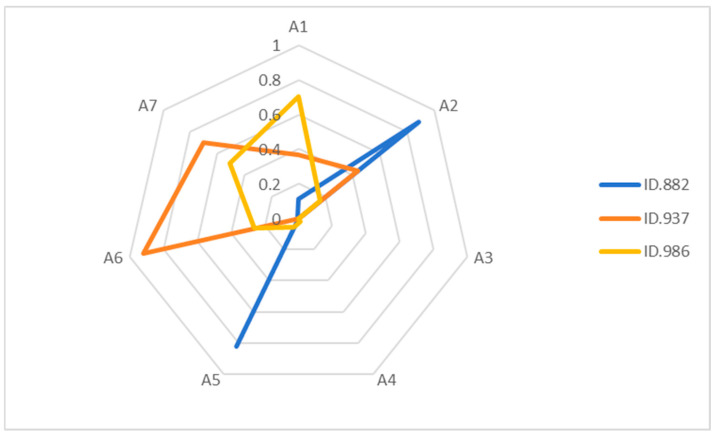
Radar Plots of Attribute Mastery for Three Students.

A key advantage of Cognitive Diagnostic Assessment lies in its ability to generate individualized knowledge profiles for each student. To demonstrate the granularity and diagnostic power of this method, we conducted an in-depth analysis of three representative students, identified as IDs 882, 937, and 986. Their radar charts (see Figure 3) vividly illustrate their distinct strengths and weaknesses across the seven cognitive attributes, offering clear guidance for personalized instruction.

As illustrated in the radar plots, the three students presented in this figure achieved identical total scores. Traditional assessment methods would consider them to be at the same proficiency level. However, from the perspective of cognitive diagnostic assessment, their mastery of cognitive attributes varies significantly, reaffirming the well-documented phenomenon of “homogeneous scores, heterogeneous cognition” (Templin and Henson 2010; De La Torre and Ma 2016).

A detailed breakdown is as follows:

Student 882 demonstrates mastery primarily in Attribute A2 and Attribute A5.

Student 937 shows strong performance in Attribute A6 and Attribute A7.

Student 986 exhibits mastery in Attribute A1 and Attribute A7.

All three students share relatively low mastery in Attribute A3, Attribute A4, and Attribute A5, which aligns with the overall pattern of these attributes being the most challenging as shown in Table 8. These findings indicate substantial differences not only in the types of attributes mastered but also in the degree of mastery. Such fine-grained diagnostic information enables truly personalized learning plans. Rather than relying solely on total scores, teachers can design tailored instructional strategies to address each student’s specific weaknesses (De La Torre 2011; De La Torre and Ma 2016; Tatsuoka 2009).

## 5. Discussion

This study leverages Cognitive Diagnostic Theory to analyze high school students’ English reading comprehension, using large-scale empirical data to systematically construct both learning paths and learning progressions that reflect cognitive sequencing and ability stratification. These findings enrich theoretical understanding and offer practical guidance for teaching and assessment.

### 5.1. Theoretical Contributions and Practical Implications

By defining and validating key cognitive attributes of reading comprehension, this study deepens the understanding of what constitutes reading proficiency. Consistent with previous research (e.g., Cai 2010), our results empirically support the presence of seven diagnostically effective attributes in the context of China’s college entrance examination. These attributes, validated with large-scale data, provide a robust foundation for modeling English reading comprehension. Furthermore, a significant theoretical contribution of this study lies in the transformation of abstract total scores into detailed cognitive profiles, revealing that students with identical total scores may possess distinctly different combinations of cognitive skills, thereby powerfully corroborating the notion of “homogeneous scores, heterogeneous cognition”. This highlights the inherent limitations of conventional testing and underscores the unique value of cognitive diagnostic assessment in providing personalized diagnostic information.

A core contribution of this study lies in its ability to characterize both linear and nonlinear aspects of learning. The findings suggest that attributes can be understood both as part of a simple easy-to-hard progression and as independent abilities. The identified “dominant learning path” represents a clear, hierarchical progression, where attributes are acquired in an order of increasing difficulty, as validated by the rising average ability scores at each step. This reflects a common, linear developmental sequence. However, the presence of multiple, less-frequent pathways in our model (see Figure 1) demonstrates that learning is not strictly linear. Some students acquire skills in a different order, reflecting more independent, non-compensatory relationships between attributes. This dual finding provides a more nuanced model of learning, acknowledging that while a typical “highway” of skill acquisition exists, various alternative “routes” are also possible, depending on individual differences in instruction and aptitude.

Moreover, this study maps students’ knowledge states to the three academic quality levels defined in the General Senior High School English Curriculum Standards (Ministry of Education of the People’s Republic of China 2020), forming a corresponding framework between micro-level cognitive sequences (learning paths) and macro-level ability tiers (learning progressions). This integration organically connects fine-grained cognitive diagnostic information with broad academic quality standards. Such a multi-level integration makes the diagnostic information more systematic and hierarchical, offering more comprehensive and meaningful feedback for students, teachers, and educational administrators. As emphasized by the U.S. National Research Council (NRC) in reports from 2005 and 2007, “learning progressions are among the most promising tools for linking curriculum standards, instruction, and assessment with students’ cognitive development to promote sustained growth” (Wei and Jia 2010). This study employs a data-driven method utilizing cognitive diagnostic assessment techniques to obtain a more objective learning progression, providing a novel perspective and a stronger scientific basis for its construction. Foster and Wiser (2012) also argue that learning progressions can significantly enhance the consistency, experiential depth, and cognitive depth of educational standards.

This research offers practical scientific tools for senior high school English teachers to implement precision education and personalized instruction. Teachers can accurately identify students’ strengths and weaknesses based on their specific positions in the learning path and corresponding levels in the learning progression, enabling the design of targeted remedial exercises. For example, students at the early stages of the learning path should first focus on developing abilities to quickly locate and extract key information from texts (i.e., Attribute A6) and to process and comprehend complex sentences (i.e., Attribute A2). For students with the knowledge state pattern (0111111), the mainstream learning path indicates that their next priority should be vocabulary expansion (i.e., mastering Attribute A1). Meanwhile, students with the knowledge state (0100011) should focus more on Attribute A3 (understanding inter-sentence relationships), which aligns better with their cognitive acquisition order. This fine-grained diagnostic feedback allows teachers to precisely arrange learning content, overcoming the limitation of traditional assessments that only provide total scores without guiding specific instructional interventions. Consequently, this facilitates truly differentiated instruction and personalized learning, significantly improving learning efficiency.

Furthermore, this study provides an empirical demonstration of the distinction between cumulative skill acquisition and the presence of isolated or ‘splinter’ skills. The analysis of the near-identical ability levels for the (1000000) and (0000010) knowledge states serves as a prime example. While A1 (‘Difficult Vocabulary’) is an advanced attribute, its mastery in isolation does not confer high overall proficiency. This has significant pedagogical implications. It cautions educators against assuming that a student’s strength in one seemingly advanced area, such as vocabulary, is indicative of overall comprehension ability. Diagnostic assessment is therefore crucial for identifying students with such unbalanced profiles. These students may require targeted instructional support not in their area of strength, but in more foundational comprehension strategies (e.g., understanding sentence structure, making inferences) to help them integrate their knowledge into a functional skill set. This highlights how fine-grained diagnostics can guide instruction in ways that holistic scores cannot.

In addition, our findings provide empirical support for optimizing curriculum design and textbook development. By linking specific cognitive attributes to the curriculum’s proficiency levels, this research validates the scientific soundness of the standard and offers feedback for future revisions. For example, the difficulty of A1 and A5 may be higher than originally classified and should be associated with higher proficiency levels. These results can inform textbook design, ensuring alignment between instructional materials and students’ actual cognitive development (Liu 2010).

In addition, the study offers scientific evidence to improve exam design and feedback reporting in high school English assessments. Exam content can be aligned with the learning path to target different cognitive levels and better differentiate student performance. Feedback reports can evolve from general total-score summaries to fine-grained diagnostic feedback, facilitating learning through assessment. This shift toward diagnostic evaluation represents a critical direction in modern educational assessment (Pellegrino et al. 2001).

### 5.2. Limitations

Despite the contributions of this study in constructing learning paths and progressions for English reading comprehension based on cognitive diagnostics, several limitations remain.

First, this study utilized cross-sectional data from a single year’s examination. While the use of the complete population dataset (N > 360,000) ensures the internal validity and stability of our findings for this specific cohort, the identified learning pathways may be subject to cohort effects or changes in curriculum over time. The decision to use the full sample, rather than a split-sample or cross-validation approach, was a deliberate choice to maximize the precision of the parameter estimates for this descriptive and inferential study. However, future research employing longitudinal tracking of student cohorts would be invaluable for validating the temporal stability and causal nature of the developmental progressions identified herein.

Second, regarding the generalizability of our findings, it is important to note the nature of our sample. While the use of the full dataset (N = 361,967) of provincial exam takers provides strong evidence for the validity of the learning pathways for this specific cohort, these findings may not be directly generalizable to other populations. Students in different educational systems, from different cultural or linguistic backgrounds, or at different age levels, may exhibit different learning trajectories. Future comparative studies could explore the stability of these pathways across diverse student samples to establish a more universal model of English reading acquisition.

Third, the construction of the Q-matrix relied on expert judgment, which, despite validation efforts, remains somewhat subjective. As the Q-matrix is central to diagnostic accuracy (De La Torre 2011), future work should explore data-driven Q-matrix validation techniques (e.g., statistical verification algorithms) to improve objectivity and precision.

Lastly, while this study proposes a detailed diagnostic reporting framework and emphasizes its instructional value, its actual impact in classrooms has not yet been empirically evaluated. The extent to which teachers and students benefit from these reports—and whether they lead to improved learning outcomes—requires further investigation. Future studies should examine changes in instructional behavior, student strategies, and long-term learning gains through experimental or action research designs (Black and William 2009).

Fourth, the dataset, while comprehensive in its inclusion of the entire test-taker population, lacked demographic information. Due to the privacy constraints associated with this high-stakes examination data, variables such as gender, age, or socioeconomic background were not provided. As a result, we were unable to explore potential differences in learning pathways or attribute mastery across various demographic groups. Future research utilizing datasets that include demographic information would provide valuable insights into the equity and differential impact of the English curriculum, helping to identify potential disparities and guiding more targeted educational interventions.

Fifth, our approach of using a single unidimensional IRT model to create the learning progression, while pragmatically useful for aligning with curriculum standards, is a simplification of the multidimensional learning process. A theoretically interesting alternative would be to model a separate IRT model for each major learning pathway identified in Figure 1. This would create multiple, parallel learning progressions, each with its own internally consistent increase in ability. Such an analysis could provide deeper insights into the proficiency trajectories of different learner subgroups and is a promising direction for future research.

## 6. Conclusions

This study employed cognitive diagnostic assessment to analyze high school students’ English reading comprehension and successfully constructed data-driven learning paths and learning progressions that reflect both micro-level cognitive sequences and macro-level proficiency levels. By diagnosing students’ mastery of specific cognitive attributes, we revealed the heterogeneity of cognitive profiles behind identical total scores, confirming the limitations of traditional summative assessments and underscoring the advantages of fine-grained, attribute-level evaluation. The dominant learning path identified in this study, validated by its wide coverage and logical cognitive sequencing, offers practical guidance for personalized instruction and curriculum design. Furthermore, by aligning knowledge states with the three proficiency levels outlined in the national curriculum, we built a coherent framework that bridges diagnostic information with educational standards.

The findings provide actionable insights for educators, curriculum developers, and policymakers. Teachers can use the diagnostic results to identify students’ strengths and weaknesses, design targeted interventions, and implement personalized teaching strategies. The learning progression model also offers a valuable reference for optimizing English reading assessments and revising curriculum standards to better reflect actual cognitive development. However, future research should validate the generalizability and longitudinal stability of the proposed learning paths using long-term tracking data. Moreover, the integration of data-driven Q-matrix validation methods and empirical studies on the practical impact of diagnostic reports will further enhance the reliability and applicability of cognitive diagnostic assessment in educational practice.

## Figures and Tables

**Table 1:** Attribute-level retest reliability.

Attribute Code.	A1	A2	A3	A4	A5	A6	A7	Mean
Reliability	0.882	0.823	0.973	0.820	0.759	0.947	0.903	0.872

**Table 2:** Cognitive Attributes for High School English Reading Comprehension.

Attribute Code	Attribute Name	Description
A1	Difficult Vocabulary	Understanding of difficult words in questions, options, or target sentences
A2	Complex Sentence Comprehension	Processing and understanding of long, semantically complex sentences and uncommon sentence patterns
A3	Understanding Inter-sentence Relationships	Understanding inter-sentence and textual coherence, including semantic and logical relationships
A4	Rhetorical and Organizational Structure Comprehension	Understanding of the organizational structure and rhetorical devices used in the text
A5	Inference	Drawing inferences based on textual information, context, and background knowledge; inferring word meanings; deducing author’s purpose, intention, and strategies
A6	Information Matching	Ability to scan and match relevant textual information to answer questions
A7	Processing of Correct Options	Accurate comprehension of questions and options, and effective elimination of incorrect options
A8	Main Idea	Understanding and summarizing the main idea of the given information

**Table 3:** Preliminary Q-matrix.

Item No.	A1	A2	A3	A4	A5	A6	A7	A8
1	0	0	0	0	0	1	0	0
2	0	0	0	0	0	1	0	0
3	0	0	0	0	0	1	0	0
4	0	0	0	0	1	0	1	0
5	1	1	0	0	0	0	1	0
6	1	1	0	0	1	0	1	0
7	0	0	0	0	1	1	1	0
8	0	0	0	0	0	1	1	0
9	1	0	0	0	0	1	1	0
10	1	0	0	0	0	1	0	0
11	0	0	0	1	1	0	1	0
12	0	0	0	1	0	0	1	1
13	1	1	0	0	0	1	0	0
14	0	0	0	0	1	1	1	0
15	0	0	0	0	0	0	1	1
16	0	0	1	0	0	0	0	0
17	0	0	1	1	0	0	0	0
18	0	0	1	0	0	0	0	0
19	0	0	1	0	0	0	0	0
20	0	0	1	1	0	0	0	0
Number of measurements	5	3	5	4	5	9	10	2

**Table 4:** Parameter Comparisons of Different Models.

Model	Number of Parameters	Deviation	AIC	BIC	RMSEA
DINA	167	7,809,984	7,810,318	7,812,122	0.06
DINO	167	7,848,767	7,849,101	7,850,904	0.071
RRUM	188	7,746,773	7,747,149	7,749,179	0.053
ACDM	188	7,742,326	7,742,702	7,744,732	0.052
LCDM	229	7,737,706	7,738,164	7,740,637	0.05
LLM	188	7,740,411	7,740,787	7,742,817	0.051
Mixed Model	219	7,659,437	7,659,875	7,662,241	0.019

**Table 5:** Wald test of 14 multi-attribute items and selected parsimonious CDMs.

Item	Selected CDMs	*p*	Item	Selected CDMs	*p*
Item4	GDINA	NA	Item11	LLM	0.2442
Item5	GDINA	NA	Item12	GDINA	NA
Item6	GDINA	NA	Item13	RRUM	0.075
Item7	GDINA	NA	Item14	LLM	0.2658
Item8	GDINA	NA	Item15	RRUM	0.7465
Item9	GDINA	NA	Item17	GDINA	NA
Item10	GDINA	NA	Item20	GDINA	NA

Note: “NA” indicates that no *p*-value was computed, as the selected model is the G-DINA model itself rather than a constrained alternative.

**Table 6:** Ability (θ) Estimates for Clustered Knowledge States.

Knowledge State	θ	Knowledge State	θ
0	−1.3357	110011	0.2508
10	−0.8406	110111	0.4526
10010	−0.215	111110	0.2566
100010	−0.4322	111111	0.6438
100011	−0.0813	1000000	−0.8412
101011	0.1528	1101011	0.3279
101110	−0.0603	1101111	0.6822
101111	0.2377	1111111	1.2282
110010	−0.3021	-	-

**Table 7:** Hierarchical Levels of the Learning Progression in High School English Reading Comprehension.

Level	Description of Academic Achievement	Attributes
1	Students can scan for and match relevant passage information for problem-solving, proficiently perform text-mapping, and process/comprehend lengthy, semantically complex, and uncommon sentence structures.	Mastery A6 and A2
2	Students can comprehend passages and questions, accurately eliminate incorrect options, comprehend inter-sentential relationships by utilizing various coherence knowledge (e.g., referential relations, ellipsis and substitution, additive relations, contrastive relations, cause–effect, temporal relations, repetition of keywords), and infer based on passage information or background knowledge.	Further mastery of A7, A3, and A5
3	Students possess an extensive vocabulary and are able to understand textual organization and rhetorical methods.	Further mastery of A4 and A1

**Table 8:** Mastery Probabilities of Each Attribute.

Attribute Code	A1	A2	A3	A4	A5	A6	A7
Attribute Mastery	0.333	0.582	0.394	0.364	0.363	0.858	0.587
Probabilities							

## Data Availability

Due to the sensitive nature of the data and confidentiality agreements with participating organizations, the raw data generated during this study are not publicly available.
